# Defining Optimal Surveillance Intervals Following Post‐Colorectal Endoscopic Submucosal Dissection

**DOI:** 10.1002/ueg2.12763

**Published:** 2025-02-17

**Authors:** Daryl Ramai, Hiroyuki Aihara, Lumir Kunovsky

**Affiliations:** ^1^ Division of Gastroenterology, Hepatology, and Endoscopy Brigham and Women's Hospital Harvard Medical School Boston Massachusetts USA; ^2^ 2nd Department of Internal Medicine – Gastroenterology and Geriatrics University Hospital Olomouc Faculty of Medicine and Dentistry Palacky University Olomouc Olomouc Czech Republic; ^3^ Department of Surgery University Hospital Brno Faculty of Medicine Masaryk University Brno Czech Republic; ^4^ Department of Gastroenterology and Digestive Endoscopy Masaryk Memorial Cancer Institute Brno Czech Republic

**Keywords:** colorectal cancer, dysplasia, endoscopic submucosal dissection, endoscopy, lesion, recurrence, resection

Endoscopic resection (ER) is indicated for the removal and management of most superficial colorectal lesions. ER which includes endoscopic mucosal resection (EMR) or endoscopic submucosal dissection (ESD) for high‐risk lesions containing dysplasia confined to the colorectal mucosa, is the most appropriate first‐line therapy and should be offered instead of surgery. The American Gastroenterological Association (AGA) recommends that patients with large complex lesions be referred to tertiary high volume centers capable of performing advanced resection by EMR or ESD [1].

Recognizing features that suggest a colorectal polyp might harbor invasive cancer is critical. Data from the Australian Colonic Endoscopic Resection study found that colorectal lesions ≥ 20 mm, a type V Kudo pit pattern, depressed component (Paris 0–IIc), complex morphology (0–Is or 0–IIa + Is Paris classification), rectosigmoid location, nongranular surface morphology, and increasing size were associated with submucosal invasive cancer [2]. To this end, it is important to recognize these features as these lesions might benefit from en‐bloc resection with ESD over piecemeal EMR.

While the topic of EMR versus ESD has been debated at length, a recent randomized clinical trial has shown ESD to be associated with significantly lower recurrence rates 1 of 161 ESDs (0.6%) and 8 of 157 EMRs (5.1%) [3]. To this end, risk factors for local recurrence following ESD include lesions ≥ 40 mm in diameter (hazard ratio [HR] 15.68 [1.88–130.5]; *p* = 0.011), piecemeal resection (HR 48.42 [10.7–218.7]; *p* < 0.001), non‐R0 resection (HR 41.05 [9.025–186.7]; *p* < 0.001), histologically incomplete resection (HR 16.23 [3.627–72.63]; *p* < 0.001), and severe fibrosis (HR 9.523 [1.14–79.3]; *p* = 0.037) [4].

Despite this, long‐term data on metachronous advanced adenoma lesions after ESD is scarce. In the recent issue of the *United European Gastroenterology* journal, Dai et al. presented a longitudinal retrospective propensity matched study of patients undergoing colorectal ESD between 2011 and 2017 [5]. The study involved 1745 subjects, with 203 post‐ESD subjects fully matched with 729 high‐risk (with 5–10 adenomas and/or advanced adenoma) and 813 low‐intermediate‐risk (1–4 non‐advanced adenomas) subjects. Among the 190 (93.6%) patients who underwent full surveillance colonoscopy 1 year after ESD, the local recurrence rate was 2.1% while the cumulative incidence of metachronous advanced lesions was 4.7%.

The cumulative incidences of metachronous advanced adenoma at 5 years in the post‐ESD, high‐risk, and low‐intermediate‐risk groups were 7.8%, 11.8%, and 5.5%, respectively. The study found that patients who underwent ESD were not associated with an increased 5‐year risk of metachronous advanced adenoma than low‐intermediate or high‐risk groups according to the US Multi‐Society Task Force guidelines. The annual risk of advanced adenoma lesions during the 5‐year period was highest in the first year post‐ESD: 4.7%, 2.0%, 0%, 0%, and 2.9%, respectively. Additionally, during the 5‐year follow‐up, no post‐colonoscopy colorectal cancer (CRC) cases were reported in the post‐ESD group, in contrast to three cases in the high‐risk group and one case in the low‐intermediate‐risk group [5].

The above data holds potential to inform clinical practice and help develop long‐term surveillance strategies. We congratulate the authors on their contribution to the literature. However, it is important to note that the study was retrospectively performed which is subject to inherent study design biases. It is worth mentioning that the authors reported an en‐bloc resection rate of 84.7% with a local recurrence rate of 2.1%. In contrast, a large Japanese multicenter prospective study involving 1740 patients undergoing colorectal ESD reported an en‐bloc resection rate of 97% with a local recurrence rare of 0.5% [6]. This study also reported metachronous invasive cancers were 0.2%, 0.9%, and 3.0% at 1, 3, and 5 years after initial ESD, respectively. To this end, the reported lower en‐bloc resection rate reported by Dai et al. may be associated with the higher local recurrence rate. Furthermore, the indication for ESD was not standardized which could influence endoscopic and clinical outcomes.

So where do we go from here? And what is the optimal surveillance recommendation following colorectal ESD? The study by Dai et al. suggested that surveillance colonoscopy after ESD can be considered at 1 year as well as 5 years (in patients without high risk factors) [5]. The study by Ohata suggested a surveillance colonoscopy 1–3 years after ESD [6]. The European Society of Gastrointestinal Endoscopy (ESGE) recommends a surveillance colonoscopy at 12 months following low (lymph node metastasis [LNM] risk < 3%) or very low (LNM risk < 0.5%–1%) risk ESD [7]. The guideline also suggests that subsequent intervals be determined according to CRC and polypectomy surveillance protocols. It is important to note that this recommendation was categorized as weak and based on low‐quality evidence. The Japan Gastroenterological Endoscopy Society (JGES) recommends that a colonoscopy should be carried out within 3 years after endoscopic treatment (level of evidence: IVb, grade of recommendation: B) [8]. However, as both studies by Dai et al. and Ohata demonstrate differences in en‐bloc and recurrence rates, this should be kept in mind when surveillance intervals are being determined. However, the above societies recognize the insufficiency of data to establish more robust clinical guidelines.

Based on our experience at a high‐referral tertiary center (Boston, Massachusetts, USA) specializing in advanced tissue resection, we generally schedule a follow‐up surveillance colonoscopy 1 year after performing a low‐risk ESD determined by en‐bloc, R0 resection case. This also includes lesions with high grade dysplasia or Tis cases.

However, it may be worthwhile to consider extending this interval to 3 years for very low‐risk patients who underwent low‐risk ESD (Figure 1). Drawing from reports mentioned above, for these very low risk‐ESD cases, it may be prudent to plan the next surveillance colonoscopy between three to 5 years, based on lesion sizes. On the other hand, for high‐risk ESD cases (which involves non‐en bloc resection or T1a curative cases), our practice has been to recommend the first surveillance colonoscopy at 3–6 months, followed by multi‐disciplinary discussions.

**FIGURE 1:**
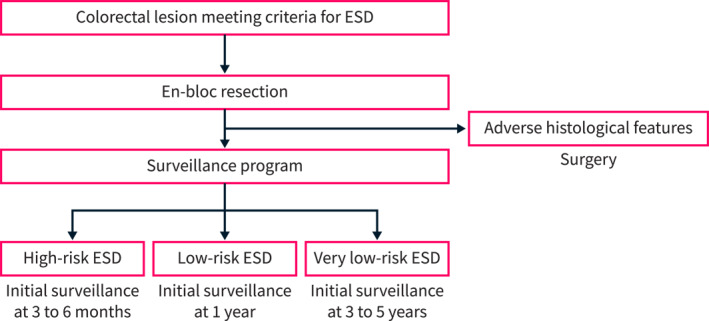
Suggested algorithm for post‐ESD surveillance. **High risk ESD:** Defined as T1a curative cases (invasion depth < 1000 μm, well or moderately differentiated, no lymphovascular invasion, and no tumor budding); and non‐en bloc resection or R1 resection (positive horizontal or deep margin) for noninvasive lesions. *Surveillance:* T1a curative cases: First surveillance in 3–6 months with subsequent intervals determined by multidisciplinary discussions. R1 for noninvasive lesions (well or moderately differentiated Tis cancers, high‐ and low‐grade dysplasia): First surveillance in 3–6 months, then intervals depend on the presence of residual lesions. **Low risk ESD:** Defined as en bloc, R0 resection of high‐grade dysplasia/well or moderately differentiated Tis cancers. *Surveillance:* 1 year, then at 3 years. **Very low risk ESD:** Defined as en bloc, R0 resection of adenomatous lesions. *Surveillance:* 3–5 years.

The expected advantage of ESD lies in its potential to reduce recurrence rates, which could result in cost savings, fewer colonoscopies, and enhanced patient care, making this topic one of utmost importance. Currently, a combination of limited research findings as well as the experience of expert endoscopists lays a framework to guide surveillance recommendations until more robust data is available.

## Conflicts of Interest

The authors declare no conflicts of interest.

## Data Availability

The authors have nothing to report.
